# Supplementary material for: Economic evaluation of Manchester procedure versus sacrospinous hysteropexy: A follow-up analysis of a randomized clinical trial
Source: PLoS One. 2025 Nov 7;20(11):e0336030. doi: 10.1371/journal.pone.0336030 (PMC12594370; doi:10.1371/journal.pone.0336030)
Supplement: S2 Fig — (PDF) [file pone.0336030.s003.pdf]

S3 Fig. Pattern of missing data on PCQ and MCQ costs

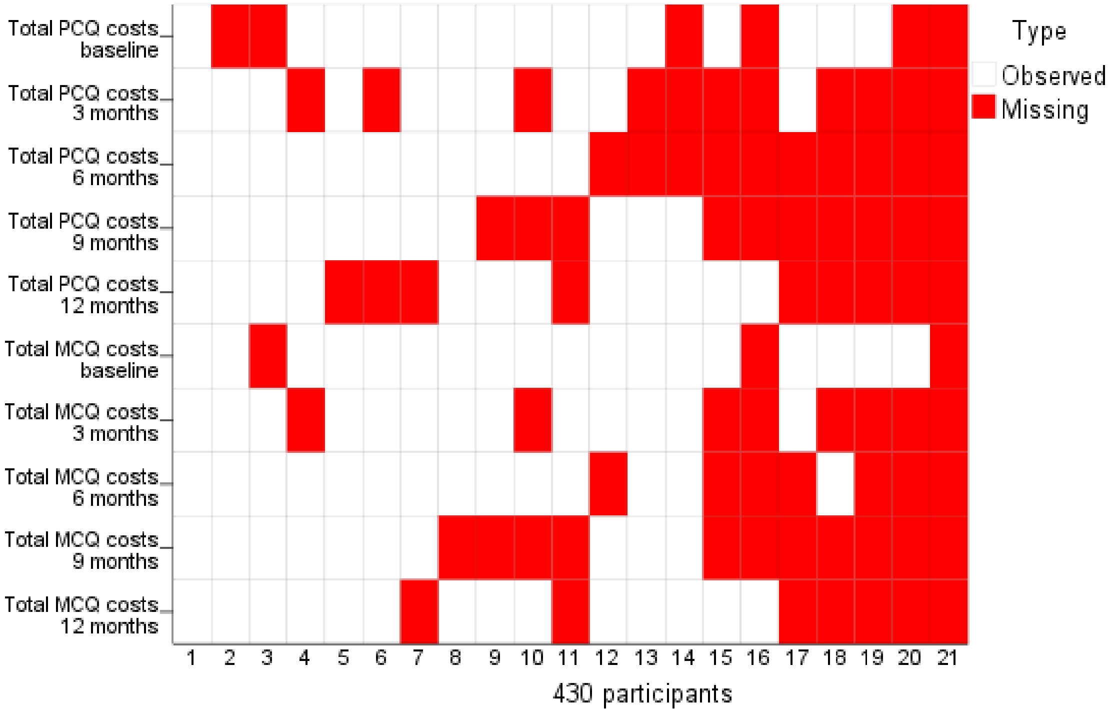

S3 Fig. Percent of cases per missing value pattern

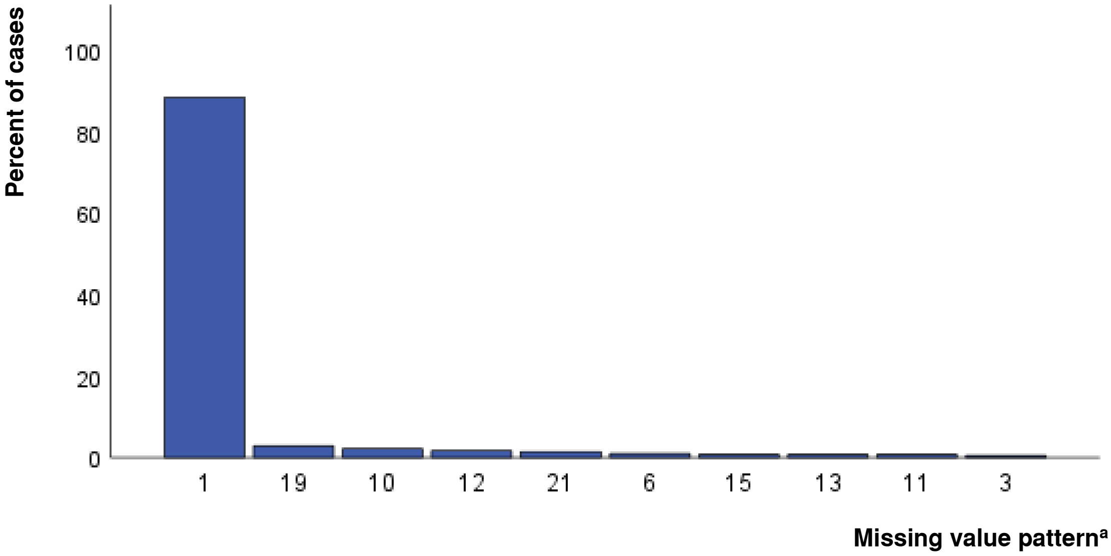

[a]10 most frequently occurring missing value patterns
