# Supplementary figures and images for: Economic evaluation of Manchester procedure versus sacrospinous hysteropexy: A follow-up analysis of a randomized clinical trial
Source: PLoS One. 2025 Nov 7;20(11):e0336030. doi: 10.1371/journal.pone.0336030 (PMC12594370; doi:10.1371/journal.pone.0336030)

S4 Fig. CE plane societal perspective

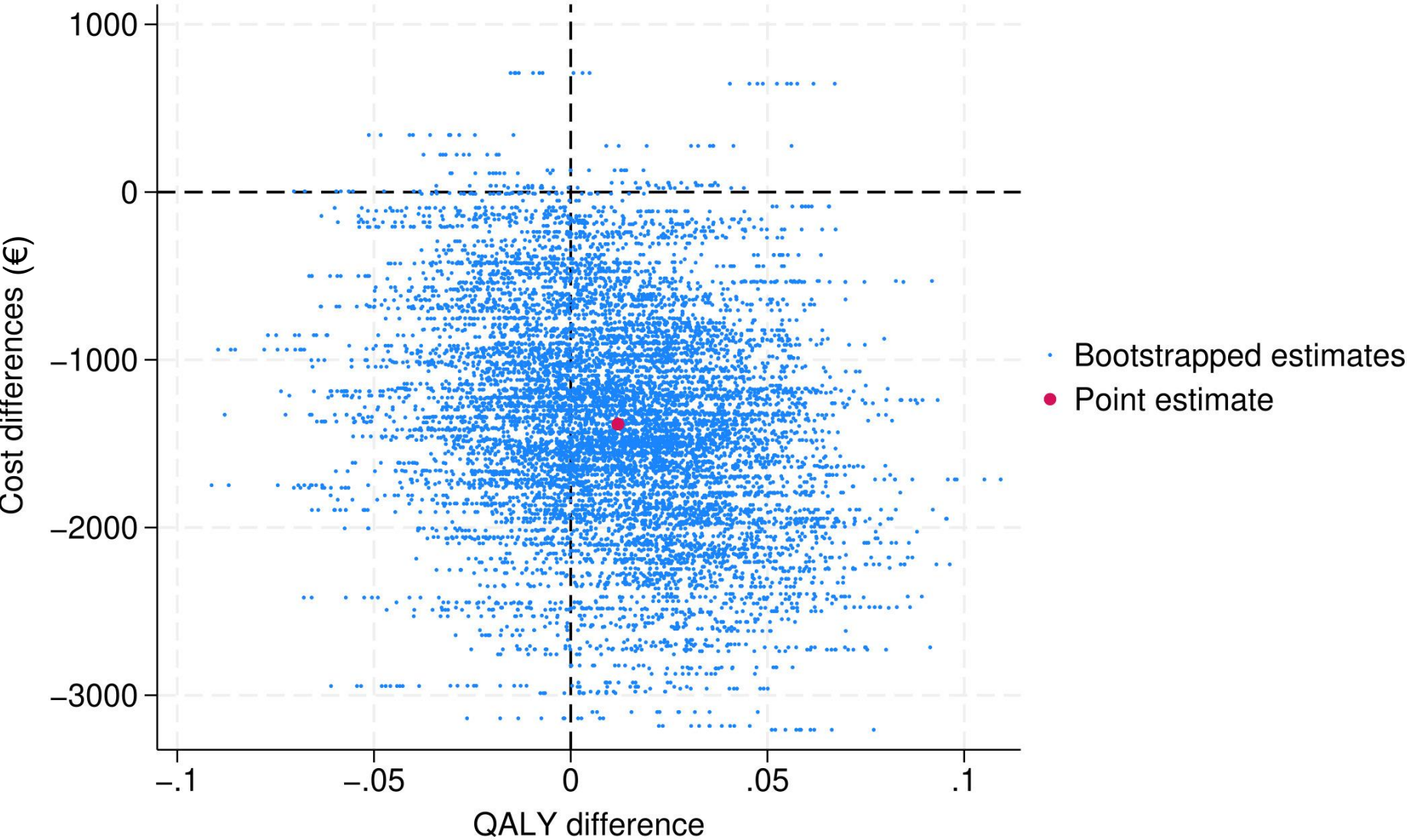

Supplement: S3 Fig — (PDF) [file pone.0336030.s004.pdf]

**S5 Fig. CE plane healthcare perspective**

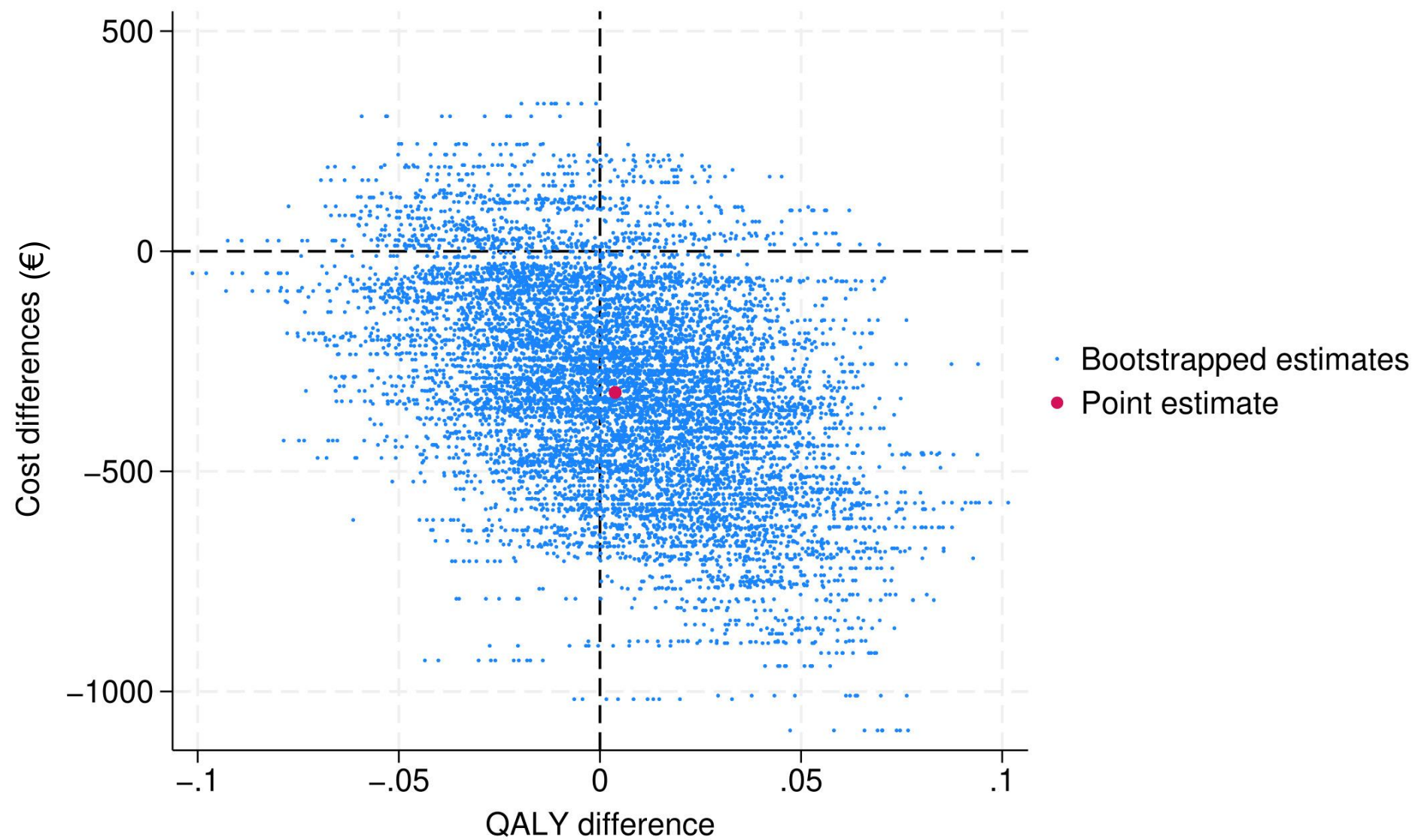

Supplement: S4 Fig — (PDF) [file pone.0336030.s005.pdf]
