# Supplementary material for: Economic evaluation of Manchester procedure versus sacrospinous hysteropexy: A follow-up analysis of a randomized clinical trial
Source: PLoS One. 2025 Nov 7;20(11):e0336030. doi: 10.1371/journal.pone.0336030 (PMC12594370; doi:10.1371/journal.pone.0336030)
Supplement: Attachment — Submitted filename: Response to reviewers 2025 05 24.docx [file pone.0336030.s007.docx]

**Academic editor comment:**

**Clarify the type of study in title to avoid confusion as it is a follow up analysis of a RCT**

Answer: Thank you for the suggestion. We have adjusted the title into: “Economic evaluation of Manchester procedure versus sacrospinous hysteropexy: a follow-up analysis of a randomized clinical trial”. (lines 1-2)

Furthermore: a short title was added to the title page: “Short title: Economic evaluation of Manchester procedure versus sacrospinous hysteropexy” (line 3)

**Journal requirements:**

When submitting your revision, we need you to address these additional requirements.

1. Please ensure that your manuscript meets PLOS ONE's style requirements, including those for file naming.

Answer: Our manuscript was now fully adjusted to meet PLOS ONE’s style requirements.

- A short title was added. (see above)

- The layout of the title page was adjusted.

- File names of supporting information were adjusted, also when used in the manuscript.

2. In the online submission form you indicate that your data is not available for proprietary reasons and have provided a contact point for accessing this data. Please note that your current contact point is a co-author on this manuscript. According to our Data Policy, the contact point must not be an author on the manuscript and must be an institutional contact, ideally not an individual. Please revise your data statement to a non-author institutional point of contact, such as a data access or ethics committee, and send this to us via return email. Please also include contact information for the third party organization, and please include the full citation of where the data can be found.

Answer: to meet the Data policy of PLOS ONE, a data repository for our dataset is now available and can be accessed openly upon publication at the following link: <https://doi.org/10.34973/wkz9-c249>. For review purposes the dataset is already available at: <https://data.ru.nl/login/reviewer-37570231/XRGPACSTMX33G7H56G64HEXLZL2KJYOE2MDH6BA>

**Reviewers comments:**

Methodological points:

1) I feel strongly that the Consolidated Health Economic Evaluation Reporting Standards 2022 (CHEERS 2022) checklist should be completed and followed: <https://www.equator-network.org/reporting-guidelines/cheers/>

Answer: The CHEERS checklist is now completed (and was already followed). We have now added the following text in the methods section of the manuscript: “The Consolidated Health Economic Evaluation Reporting Standards 2022 (CHEERS 2022) checklist was followed (S1 appendix).” (lines 118-119)

2) More detail is needed around missing data: for example, how many imputations were used in the multiple imputation?

Answer: Missing data were assumed to be missing at random. We used Multiple Imputation by Chained Equations (MICE) with predictive mean matching. We used 10 imputed datasets (here the loss of efficiency was sufficiently small <5%). We have now added the following text in the methods section of the manuscript: “Multiple imputation by chained equations with predictive mean matching was applied to impute the missing values and ten data sets were created. All available baseline characteristics, operative variables and outcome data were used for imputation.” (lines 148-151)

3) What were the patterns of missing data? Please review Faria et al 2014 and follow its suggestions: <https://pubmed.ncbi.nlm.nih.gov/25069632/>
   At minimum, I would like to see some plots of missing data in supplementary materials.

Answer: We now added figures to the supplementary material that show the pattern of missing data for EQ-5D-5L, MCQ and PCQ at different measurements. We added the following text to the results section: “See S2 Fig 1 for patterns of missing data on EQ-5D-5L and S2 Fig 2 for patterns of missing data on MCQ and PCQ at different measurements.” (line 178-179)

4) How did you combine bootstrapping with the multiple imputation? Did you bootstrap from each imputed data-set?

Answer: The imputed datasets were analyzed separately to obtain a set of estimates which were then pooled using Rubin’s rule to obtain overall estimates, standard errors, and confidence intervals.

5) Regression models: did you control for any other variables? Was there any stratification in randomisation of the RCT? If there was, these variables should be included in your regression models. Did you adjust for site? Generally speaking, I would try to follow the statistical approach adopted in the RCT as far as possible – they may used a mixed effects model, which may preclude a SUR model.

Answer: We did not control for other variables and no stratification was applied. The randomization in the original trial was successful. No adjustment for sites was applied. In the original trial we did not use a mixed effect model. The statistical analysis plan of the original trial can be found here: <https://cdn.jamanetwork.com/ama/content_public/journal/jama/939206/joi230086supp2_prod_1694540303.2364.pdf?Expires=1751255676&Signature=ROriEjhYV9ZJ9wno1NhUYcmAiuK-n3-a4xTe7FG7KjkEr53fCNTXW1YVnXSncLYSpMU8rzgAZAWif~E4z2rD3JurPGuOyx8w5OnIahyzni3rOyJxz2aMfvdgtswHMNAM8oNdoD6d82jcmfm3qMQX~qoNNrOp0BrtTTjygQEoXR42ODtLsSIxBwS5rMGhNzr2hedji2m~Ruv7yyIuY0FI0qgdwQJqYcxAa-P7OX-kPk0EL0mD3K4WDLv~MPIhw2vDu7w1y9if5Y~5uv~i0dXW5IrbFD2tRiLS22f5JXv2lPJElxPcLjazK~pjN4uDu6Mo9vqf28ctMEPinK7L5bdMwg__&Key-Pair-Id=APKAIE5G5CRDK6RD3PGA>.

This document together with the study protocol are also available upon publication in the open access data repository: <https://doi.org/10.34973/wkz9-c249>.

6) L122-124 – what valuation set was used to turn EQ-5D scores into utilities?

Answer: The Dutch tariff for the EQ-5D-5L was used. We now added the following text to the methods section: “The EQ-5D-5L Dutch tariff was used to calculate utilities.” (line 144)

7) Was discounting used for the second year of costs and effects? (May depend on the Dutch guidelines)

Answer: Given the relatively short evaluation period of two years as well as we assume that in this short-term empirical CEA, no discounting improves generalizability across countries, it was decided not to discount in the complete case scenario. However, we mention the impact of discounting according the Dutch guidelines [1] for both societal costs and QALY’s and included this in the results. The discounting effect was not statistically significant for both the variables:

|  | **No discount** | | | **Discount** | | |
| --- | --- | --- | --- | --- | --- | --- |
|  | SSH | Manchester | Mean difference | SSH | Manchester | Mean difference |
| QALY | 1.69 | 1.70 | 0.01 (-0.05 to 0.07) | 1.66 | 1.67 | 0.01 (-0.05 to 0.07) |
| Societal costs | 70 | 78 | -8 (-138 to 121) | 66 | 74 | -8 (-130 to 114) |

We now added the following text to the methods section: “In the complete case scenario no discounting was applied for improving generalizability across countries.” (lines 157-159) Furthermore we added the following text to the results section: “Discounting did not have a significant influence on these results.” (lines 191 and 210)

8) Supplementary tables with finer detail of resource and cost break down is needed – more detail is needed than just table 4 – perhaps one table, with a row for each “unit cost” resource, comparing arms. Should be repeated for costs.

Answer: Unit cost resources were the same for both arms (the operation itself excluded), and were therefore shown in a table with one column (table 1). We have now added a cost break down in table 4 for the different types of costs that were included in the analyses.

9) Precision: Table 4 and more generally: I am very unconvinced that clarity is provided by including cents – why not round costs to the nearest euro? Could you use a thousands separator?

Answer: costs were now rounded to the nearest euro and a thousand separator was used in table 1 and 4.

10) Figures 1-4: I am unconvinced you need both the CE planes and CEACs – why not move the CE-planes to supplementary materials? Also, I think it is a waste of space to have separate figures for the CEACs – why not one plot, with two lines?

Answer: We thank the reviewer for this good suggestion. The CE planes are now moved to the supplementary material. Furthermore, a CEAC with two lines was created and is now named Fig 1. Furthermore, the text was adjusted in the results section, so it fits to the new figures and supplementary materials: “S3 Fig 1 and S3 Fig 2 show the CE planes of the societal perspective and health care perspective, respectively, presenting the bootstrapped incremental CE ratios. In the CE plane for societal perspective, 65% of the points are in the South-East quadrant. In the CE plane for health care perspective, 51% of the points are in the South-East quadrant. It shows that sacrospinous hysteropexy and the Manchester procedure have comparable effectiveness expressed as QALYs gained, but sacrospinous hysteropexy is more expensive.“ (lines 230-235)

11) L213: “the procedures showed equal effectiveness expressed in QALYs” – I would advise caution with this statement – I think some statisticians would argue that you have not done a non-inferiority study on comparing procedures, so can’t claim equal effectiveness – rather, you have found no statistically significant evidence of difference – the arguments are not reversible. I tend to follow Claxton 1999 (<https://doi.org/10.1016/s0167-6296(98)00039-3>) on this point, and would focus on the point estimates and the certainty of findings.

Answer: The word ‘equal effectiveness’ was now replaced for ‘no statistically significant different effectiveness’ or ‘we found no statistically significant evidence of difference’ throughout the manuscript. (lines 47, 242-243) The word ‘equal’ was replaced for ‘similar’ in the discussion section. (line 306)

More minor points:

12) L21, Abstract: “Common condition”– include some numbers to speak to this?

Answer: We now added the following text to the abstract: ‘The reported lifetime risk of undergoing pelvic organ prolapse surgery is estimated to affect up to 20% of women.’ (lines 24-25)

13) L23, Abstract: “As the costs in healthcare are rising, the question raises whether an economic evaluation likewise points out in the direction of the Manchester procedure.” – I found this hard to parse – perhaps something like: “It is also important to consider the resource and associated cost implications of the choice between these two procedures – we conduct an economic evaluation to compare the alternative costs and benefits.”

Answer: We understand the reviewer’s suggestion. The sentence was replaced with the following text in the abstract section: “it is also important to consider the resources and associated cost implications of the choice between these two procedures. An economic evaluation was conducted to compare the alternative costs and benefits.” (lines 27-29)

14) L61/62: I’m unconvinced the main point is that healthcare costs are rising (not that I disagree) – I would just make the point you want to do the best with available resources – as argued in L63 – perhaps particularly relevant if the healthcare system is publicly funded.

Answer: We have now adjusted the paragraph to make the suggested point. “Cost-effectiveness (CE) analyses are needed to provide the most appropriate care with available resources. This is particularly relevant in countries with a publicly funded health care system.” (lines 68-71)

15) L69: First mention of “SAM study” - should be spelt out/introduced.

Answer: The following text was now added to the introduction: “SAcrospinous hysteropexy versus the Manchester procedure”. (line 77)

16) Table 1: I find the “units” column unhelpful – I would suggest absorbing the related detail in the resource label, or the unit price – e.g. for the latter, in terms of travel costs – “0.19/km” etc

Answer: The unit column was removed from table 1 and a footnote is now added to table 1, explaining the unit prices. ‘Per hour’ and ‘per kilometer’ is now added to the resources column. The following text is added to the footnote: “Prices are presented per 1 unit unless stated otherwise. Reference prices were based on the Dutch guideline for healthcare cost analysis. TVT=tension-free vaginal tape; km=kilometer.”

17) Table 1: The unit costs introduce the idea of “re-intervention” and complications – these should be motivated/covered when the procedures are introduced/perhaps in the background. For example, if randomised to the Manchester procedure and a re-intervention is necessary, which of the Manchester procedure or Sacrospinous would be used?

Answer: Unfortunately, there is no clear guideline or flowchart for the re-interventions in case of prolapse recurrence and complications. The type of re-intervention depends on the pelvic floor problem(s) the women encounters and her personal preference. This can vary widely and cannot be estimated in advance. We have now added the following text to the methods section: “In the event that a woman experiences symptoms of recurrence, such as a sensation of bulging, a re-intervention may be considered. The decision to proceed with re-intervention depends on the pelvic floor symptoms, anatomy and stage of the recurrent prolapse, as well as the woman's preferences. Re-intervention options include pessary treatment or surgery. In case surgery is needed after sacrospinous hysteropexy or the Manchester procedure, the options include anterior and/or posterior colporrhaphy, perineoplasty, vaginal hysterectomy, sacrospinous hysteropexy or the Manchester procedure, (robot-assisted) sacral colpopexy, and/or tension-free vaginal tape (TVT). There is no reliable data available regarding the number of women who undergo specific procedures as a second intervention.” (lines 94-103)

18) Table 5: It would seem natural to extend the table with QALYs at 1 year, 2 years and in total.

Answer: We thank the reviewer for this suggestion. We have now added the QALYs at 1 year, 2 years and in total to table 5.

19) L192, L196: To convey some of the uncertainty etc, would it be useful to report the percentage of points falling in the South-East (more-effective, less costly) quadrant of the CE plane?

We now added the following text to results section: “In the CE plane for societal perspective, 65% of the points are in the South-East quadrant. In the CE plane for health care perspective, 51% of the points are in the South-East quadrant.” (lines 231-233)

20) L218-233: You assert that the important costs and consequences are captured in two years, though others have used models, which I assume have a longer time horizon than 2 years – please add a sentence or two to address this – what was the time horizon in these models? Why didn’t they stop at two years? Etc More broadly, in your literature overview, I think it would be useful to capture the time horizon of these models – is there any “correlation”/link between the time horizon and positive/negative CE findings?

Answer: The studies that are referenced in our manuscript as the available literature are not on original studies on costs, but they describe models based on clinical studies on effectiveness. These models have indeed used a timeframe of 5 years, which is longer than in our study. Most of the studies underlying the models, however, do not have a follow-up duration of 5 years. (In the study on the model by Wang et al., it is stated that the model is based on a meta-analysis of 106 outcome studies. The mean follow-up of these studies differs between the types of pelvic floor surgery, and varies between 17.2 and 32.6 months.). We are unsure why these studies describing models have used a time frame of 5 years. Most re-operations occur during the first 2 years after surgery, and this thereby seems a feasible and reasonable follow up period to us. For clarity, we added the following text to the discussion section: “with a time horizon of 5 years”(line 249) and “Abovementioned studies had longer time horizons than our study, which could put results in a different perspective.” (lines 263-264)

Very minor points:

21) L65: Perhaps replace: “likewise points out in the direction of” -> “similarly favours”

Answer: The text was adjusted to ‘similary favors’ (American English) (line 73-74).

22) L75: Replace “in case” with “if”?

Answer: The text was adjusted to ‘if’ (line 83).

23) L103: eCRF needs spelling at first use.

Answer: “electronic Case Report Form” is now added to line 102.

24) L109: I think pluralise “unit cost” – more than one unit?

Answer: the sentence is now pluralized (line 120).

25) Table 1: TVT needs spelling out

Answer: TVT is now spelled out in the footnote.

26) L121: I believe more standard for EQ5D to be capitalised as “EQ-5D-5L”, as per the EuroQol website: <https://euroqol.org/information-and-support/euroqol-instruments/eq-5d-5l/>

Answer: “EQ-5D-5L” is now used throughout the manuscript.

27) L139: It seems odd to me that data extraction from CastorEDC are mentioned here – why not move this sentence to L102-108 etc?

Answer: The line has now been moved to the suggested paragraph (line 126-127).

28) Table 2: You include variables that are not baseline characteristics (e.g. primary outcome and reintervention etc) – please update the caption. Would some of these variables sit better in Table 3?

Answer: the variables have now been moved to table 3.

29) L200: Should “if” be “as”?

Answer: “if” has now been replaced with “as” (line 228).

The following sentences were rewritten for better readability:

Lines 282-284: The sentence “Furthermore, we had expected repeat surgery for prolapse would cause more evident differences in costs, but repeat surgery alone was not determinative.” was amended into: “Moreover, we anticipated that repeat surgery for prolapse would result in more pronounced cost differences; however, repeat surgery alone was not a decisive factor."

Lines 305-308: two sentences have been split by the word ‘furthermore’ for better readability: “However, logically, costs regarding the primary surgery (including revalidation and treatment of complications) are considered to be incurred within the first year after surgery. Furthermore, data on repeat surgery for recurrences, which may be incurred in the second year as well, are less likely to be prone for recall bias.”

**References**

1.	Richtlijn voor het uitvoeren van economische evaluaties in de gezondheidszorg (versie 2024) 2024 [cited 2025 Apr 17].
