# Supplementary material for: Economic evaluation of Manchester procedure versus sacrospinous hysteropexy: A follow-up analysis of a randomized clinical trial
Source: PLoS One. 2025 Nov 7;20(11):e0336030. doi: 10.1371/journal.pone.0336030 (PMC12594370; doi:10.1371/journal.pone.0336030)
Supplement: Attachment — Submitted filename: Response to reviewers 2025 08 14.docx [file pone.0336030.s008.docx]

**Response to reviewers**

**Journal requirements:**

Please review your reference list to ensure that it is complete and correct. If you have cited papers that have been retracted, please include the rationale for doing so in the manuscript text, or remove these references and replace them with relevant current references. Any changes to the reference list should be mentioned in the rebuttal letter that accompanies your revised manuscript. If you need to cite a retracted article, indicate the article’s retracted status in the References list and also include a citation and full reference for the retraction notice.

The reference list was reviewed to identify retracted publications; no such papers were found. Several references were updated:

- Reference 14 was revised to conform to the PLoS Vancouver citation style:

Consumer prices; price index 2015=100 [Internet]. CBS. 2022 [cited 2022 Mar 9]. Available from: https://www.cbs.nl/en-gb/figures/detail/83131ENG

- Reference 16 was updated with the most recent version of the source and a hyperlink was added:

Hakkaart-van Roijen L, Peeters S, Kanters T, van Baal P, Brouwer W, Drost R, et al. Kostenhandleiding voor economische evaluaties in de gezondheidszorg [Report]. Diemen: Zorginstituut Nederland; 2024 [cited 2022 Mar 9]. Available from: https://www.zorginstituutnederland.nl/over-ons/publicaties/publicatie/2024/01/16/richtlijn-voor-het-uitvoeren-van-economische-evaluaties-in-de-gezondheidszorg

- Reference 17 was corrected to align with the PLoS Vancouver style:

Medicijnkosten.nl [Internet]. Zorginstituut Nederland (ZIN). 2023 [cited 2022 May 23]. Available from: https://www.medicijnkosten.nl/

- Reference 23 was updated to reflect the correct publication details:

Zorginstituut Nederland. Richtlijn voor het uitvoeren van economische evaluaties in de gezondheidszorg [Internet]. Diemen: Zorginstituut Nederland; 2016 [cited 2020 Aug 25]. Available from: https://www.zorginstituutnederland.nl/documenten/2024/01/16/richtlijn-voor-het-uitvoeren-van-economische-evaluaties-in-de-gezondheidszorg*

**Editor Comments:**

Thank you for submitting your manuscript to PLOS ONE. After careful consideration, we feel that it has merit but does not fully meet PLOS ONE’s publication criteria as it currently stands. Therefore, we invite you to submit a revised version of the manuscript that addresses the points raised during the review process.

**Review comments**

**Reviewer #2**

Positives:
The title adequately describes the study
The Economic evaluation of Manchester procedure versus sacrospinous hysteropexy" is a comprehensive assessment comparing the alternative costs and benefits of different healthcare procedures
The CHEERS 2022 checklist was followed
Methods used to asses direct costs , indirect costs and QALYs are all standard
Conclusions reached are representative barring a few concerns

Negatives

1. The 2-year follow-up is too short to reflect long term societal costs based on the definition of failure in this paper [mass symptoms which requires pessary/ surgical intervention]. As all mass symptoms do not require surgery and the progressive nature of prolapse which can worsen over time. Longer follow up of at least 5 years is often needed to capture ongoing treatment costs, disease progression, and long-term economic consequences as stated in the conclusion.

Thank you for your review of our paper and your valuable comments.

Balancing feasibility and ambition, we believe that the follow-up period in this study is adequate and allows us to present these findings as relevant and meaningful data. As a cost-effectiveness analysis is inherently incremental, it focuses on differences in costs and QALYs that are most relevant. Within the first two years after surgery, cost differences—particularly those related to productivity—are most pronounced. Given the decreasing incremental cost differences between the procedures over time, we are confident that this timeframe captures the most critical period of cost development.

After two years, both groups tend to converge in terms of healthcare utilization and, indirectly, productivity-related costs. This convergence is also reflected in quality of life outcomes, which are largely comparable between groups at the two-year mark. The observed cost difference of 1458 euros per patient suggests that these differences are likely to remain meaningful, even with a longer follow-up period. While individual patients may experience long-term consequences of surgical treatment for prolapse, these are not likely to result in substantial changes in the cost differences between the groups.

2. Kindly explain how all relevant cost and effect differences were calculated if [Line 96-97] there is no reliable data available regarding the number of women who undergo specific procedures as a second intervention how can costs be calculated.

We thank the reviewer for this valuable comment. We acknowledge that our explanation in the manuscript may have lacked clarity. We do know exactly which repeat procedures have been performed within the 2 year follow-up of the SAM study, and we have included those costs. There is however no standard protocol on best treatment in cases of recurrence after a sacrospinous hysteropexy or Manchester procedure. We have now clarified this in the paper: “Currently, there is no standard protocol on the best treatment in case of recurrence after a sacrospinous hysteropexy or Manchester procedure.” (lines 94-95)

3. To conclude [line 233-34]that the recurrence rates are lower than other comparative studies 2yr vs 5 year follow up is erroneous.

We thank the reviewer for this comment. The sentence could indeed lead to confusion. We adjusted the sentence for clarity: “In the SAM study, the thresholds for repeat surgery were not reached within two years postoperatively, and it is unlikely that they will be reached at the five-year follow-up. Data for this extended follow-up are currently being collected.” (lines 233-235)

**Reviewer #3**

The present issue places the consequence of the SAM study, which concluded that sacrospinous hysteropexy is inferior to the Manchester procedure.
Through the present articles, sacrospinous hysteropexy is not entirely inferior to Manchester procedure, taking into consideration about the QALYs.

Actually, almost half of participants belong to the POP-Q 2nd degree. Strictly speaking, those patients tend to be cured even by the use of Pessary, the cheapest procedure. However, this time the highlight is the comparison between sacrospinous hysteropexy and Manchester procedure and not focused on the other procedures.

So far as this second revised articles, there are no suspicious points for me. I really respect your hard works.

Thank you for your thoughtful reflection and kind compliments. We truly appreciate your engagement.
