# Supplementary material for: Economic evaluation of Manchester procedure versus sacrospinous hysteropexy: A follow-up analysis of a randomized clinical trial
Source: PLoS One. 2025 Nov 7;20(11):e0336030. doi: 10.1371/journal.pone.0336030 (PMC12594370; doi:10.1371/journal.pone.0336030)
Supplement: Attachment — Submitted filename: review_PLOS_sslf_2025.docx [file pone.0336030.s009.docx]

**Article review**

Overall comment:

1. Overall, the study is well-written and flows smoothly. The findings and the authors’ intentions are clear and easy to follow. I appreciate the hard work involved and am impressed by the initial effort that went into designing and completing the SAM study. Based on its results, it is evident that the Manchester procedure is more cost-effective, primarily due to its lower recurrence rate. This naturally may make the readers wonder what the composition of reoperations is, but it is not critical. While the actual costs may not precisely reflect those in other countries (particularly the U.S.), the analysis provides useful context for cost considerations in that setting.
2. The authors have also adequately revised the manuscript in response to the reviewers’ previous comments; however, I have added a few more that need answering.
3. Great spin-off of the initial RCT.

Specific comments:

1. Line [63-66] – This statement feels somewhat overstated, even though I recognize it is based on the SAM study results. I suggest using less definitive language than “now that xx is proved to be,” since the Manchester procedure is not currently regarded as the standard of care for all vaginal POP procedures.

Materials and methods:

1. Clear and well-structured explanation of the materials and methods; acceptable with no major issues noted.

Economic evaluation:

1. The authors’ response to Reviewer #2 regarding the study duration is adequate; however, they could elaborate further than what is currently provided in lines [108–109] to help address potential public concerns when the study is published.
2. **Table 1:** Why is the re-intervention cost for SSLF/Manchester lower than the primary surgery?
3. Please consider adding a comment on how more recent data, particularly following the COVID-19 pandemic, might influence the findings—whether it would diminish or amplify the cost evaluations based on decade-old data—even if the overall conclusion remains unchanged.
4. Additionally, have the authors considered detailing the types of reoperations performed? While not critical, there remains a question as to whether patients in the SSLF group required more “costly” subsequent surgeries. If referenced in the initial SAM study, could reference it.

Results:

1. Table 3, Urinary retention with treatments: the numbers of “treatments” do not add up as well as the %.

Costs:

- A minor suggestion: consider moving lines [183–185] to follow the first paragraph and place them above Table 4 to improve the overall flow of the study.
