# Supplementary material for: Economic evaluation of Manchester procedure versus sacrospinous hysteropexy: A follow-up analysis of a randomized clinical trial
Source: PLoS One. 2025 Nov 7;20(11):e0336030. doi: 10.1371/journal.pone.0336030 (PMC12594370; doi:10.1371/journal.pone.0336030)
Supplement: Attachment — Submitted filename: Response to reviewers 2025 10 12.docx [file pone.0336030.s010.docx]

**Response to reviewers**

Overall comment:

1. Overall, the study is well-written and flows smoothly. The findings and the authors’ intentions are clear and easy to follow. I appreciate the hard work involved and am impressed by the initial effort that went into designing and completing the SAM study. Based on its results, it is evident that the Manchester procedure is more cost-effective, primarily due to its lower recurrence rate. This naturally may make the readers wonder what the composition of reoperations is, but it is not critical. While the actual costs may not precisely reflect those in other countries (particularly the U.S.), the analysis provides useful context for cost considerations in that setting.
2. The authors have also adequately revised the manuscript in response to the reviewers’ previous comments; however, I have added a few more that need answering.
3. Great spin-off of the initial RCT.

Thank you for your thoughtful comments.

Specific comments:

1. Line [63-66] – This statement feels somewhat overstated, even though I recognize it is based on the SAM study results. I suggest using less definitive language than “now that xx is proved to be,” since the Manchester procedure is not currently regarded as the standard of care for all vaginal POP procedures.

The word ‘proved’ has now been amended into ‘appears’. [line 64]

Materials and methods:

1. Clear and well-structured explanation of the materials and methods; acceptable with no major issues noted.

No amendments have been made.

Economic evaluation:

1. The authors’ response to Reviewer #2 regarding the study duration is adequate; however, they could elaborate further than what is currently provided in lines [108–109] to help address potential public concerns when the study is published.

The following sentence has been added with three new references: “These costs include rehabilitation after surgery, management of complications, and treatment of recurrences. This is supported by studies indicating that most recurrences occur within the first two postoperative years.” [line 109-111]

2. **Table 1:** Why is the re-intervention cost for SSLF/Manchester lower than the primary surgery?

The observed cost differences are attributable to the inclusion of anterior and/or posterior colporrhaphy procedures within the primary surgery costs. As no re-intervention involving sacrospinous hysteropexy was conducted, the corresponding row has been removed from the table. No further amendments were made.

3. Please consider adding a comment on how more recent data, particularly following the COVID-19 pandemic, might influence the findings—whether it would diminish or amplify the cost evaluations based on decade-old data—even if the overall conclusion remains unchanged.

The SAM trial was partly conducted during the pandemic. Many of the procedures were postponed as a result of pandemic related disruptions. However, we do not believe this had a significant impact on the overall costs. Current costs are likely higher due to inflation. However, we like to emphasize that CEA is incremental with regard to analysis by nature, so the difference will relatively be unaffected especially as the randomized clinical trial had a well balanced inclusion regarding control and intervention arm. So both groups met the same COVID-19 challenges. No amendments were made.

4. Additionally, have the authors considered detailing the types of reoperations performed? While not critical, there remains a question as to whether patients in the SSLF group required more “costly” subsequent surgeries. If referenced in the initial SAM study, could reference it.

We thank the reviewer for this suggestion. In table 4 extra costs are mentioned, which include the average cost per participant for repeat surgery (among others). Although the cost difference between the interventions is statistically significant, it constitutes only a minor component of the overall disparity in societal costs. Therefore, we have not detailed the reoperations. The significant difference in societal costs is mainly due to the productivity losses. In the table below, the details of the reoperations are shown.

|  | **Sacrospinous hysteropexy**  **(n=207)** | **Manchester procedure**  **(n=213)** |
| --- | --- | --- |
| **Repeat surgery for POP** | 11/207 (5.3%) | 4/213 (1.9%) |
| Repeat surgery operated compartment | 9/207 (4.3%) | 0 |
| Repeat surgery non-operated compartment | 2/154 (1.3%) | 4/144 (2.8%) |
| **Type of repeat procedure** |  |  |
| Manchester procedure | 3 (1.5%) | 0 |
| Cervical amputation | 2 (1.0%) | 0 |
| Posterior colporrhaphy | 2 (1.0%) | 2 (0.9%) |
| Anterior colporrhaphy | 3 (1.5%) | 1 (0.5%) |
| Vaginal hysterectomy | 1 (0.5%) | 0 |
| Robot assisted sacropexy | 4 (1.9%) | 1 (0.5%) |
|  | | |

Results:

1. Table 3, Urinary retention with treatments: the numbers of “treatments” do not add up as well as the %.

Table 3 has been adjusted, for clarity another line has been added in the table with the number of women who received both a foley catheter and self-catheterization.

Costs:

- A minor suggestion: consider moving lines [183–185] to follow the first paragraph and place them above Table 4 to improve the overall flow of the study.

The manuscript was amended accordingly.
